# Wild boar mapping using population-density statistics: From polygons to high resolution raster maps

**DOI:** 10.1371/journal.pone.0193295

**Published:** 2018-05-16

**Authors:** Claudia Pittiglio, Sergei Khomenko, Daniel Beltran-Alcrudo

**Affiliations:** Animal Production and Health Division, Food and Agriculture Organization of the United Nations, Viale delle Terme di Caracalla, Rome, Italy; U.S. Geological Survey, UNITED STATES

## Abstract

The wild boar is an important crop raider as well as a reservoir and agent of spread of swine diseases. Due to increasing densities and expanding ranges worldwide, the related economic losses in livestock and agricultural sectors are significant and on the rise. Its management and control would strongly benefit from accurate and detailed spatial information on species distribution and abundance, which are often available only for small areas. Data are commonly available at aggregated administrative units with little or no information about the distribution of the species within the unit. In this paper, a four-step geostatistical downscaling approach is presented and used to disaggregate wild boar population density statistics from administrative units of different shape and size (polygons) to 5 km resolution raster maps by incorporating auxiliary fine scale environmental variables. 1) First a stratification method was used to define homogeneous bioclimatic regions for the analysis; 2) Under a geostatistical framework, the wild boar densities at administrative units, i.e. subnational areas, were decomposed into trend and residual components for each bioclimatic region. Quantitative relationships between wild boar data and environmental variables were estimated through multiple regression and used to derive trend components at 5 km spatial resolution. Next, the residual components (i.e., the differences between the trend components and the original wild boar data at administrative units) were downscaled at 5 km resolution using area-to-point kriging. The trend and residual components obtained at 5 km resolution were finally added to generate fine scale wild boar estimates for each bioclimatic region. 3) These maps were then mosaicked to produce a final output map of predicted wild boar densities across most of Eurasia. 4) Model accuracy was assessed at each different step using input as well as independent data. We discuss advantages and limits of the method and its potential application in animal health.

## Introduction

The wild boar (*Sus scrofa*, L. 1758) is a generalist and opportunistic species found from western Europe and the Mediterranean Basin to eastern Russian Federation and Japan, throughout southeast Asia. Wild boar occupies one of the largest geographic range among all terrestrial mammals [1,2], including a variety of habitats, vegetation types and climate. Due to a combination of biological (i.e., species ecological plasticity, high reproduction rate), environmental (i.e., climate change, mild winters), and anthropogenic factors (i.e., depopulation of rural areas, reintroduction, lack of large natural predators, change in agricultural practices, reduced hunting pressure, supplementary feeding and other husbandry practices), the abundance of the wild boar has continuously increased over the last decades and its distribution expanded across the whole geographic range [3,4,5,6]. This expansion poses a threat to the agriculture, conservation and livestock health sectors, as the wild boar is an invasive and pest species causing substantial economic loss [7,8]. In recent years, there has been an increase in the likelihood of disease spread, car accidents, damages to crops and to natural vegetation [4]. In particular, the species may represent a reservoir or play another type of role in the transmission of many livestock, wildlife and human diseases such African and classical swine fever, brucellosis, tuberculosis, salmonellosis, Aujeszky’s disease and foot and mouth disease [9]. Domestic pigs and wild boar share most diseases. A review of viral diseases of the European wild boar with a direct effect on wild boar and an economic impact on domestic pig production systems identified 17 viral agents [9]. The involvement of the species in the recent expansion and persistence of African swine fever in eastern Europe and the Caucasus, despite control efforts, has attracted considerable international attention, stressing the difficulties of managing animal diseases in wild populations [10].

The management and control of wild boar populations require accurate and detailed spatial information on species distribution and abundance. Wild boar density varies from 0.01 to 43 animals / km^2^, following an east-west biogeographical gradient and exhibiting high inter-annual fluctuation due to their high reproductive potential [2,11]. However, accurate and detailed wild boar population statistics, such as census and hunting data, are not available for large-scale studies [11,12,13,14]. Two recent studies have extrapolated and predicted wild boar distribution and expansion at global level using wild boar data available across parts of the geographical range of the species [5,6]. This is probably due to the ecological traits of the species (e.g. complex social structure, nocturnal activity pattern, preference for dense vegetation, and high inter- and intra- annual variability in reproduction rate), that make direct observations over large geographic areas more difficult and expensive than the collection of indirect signs, such as faecal droppings, tracks, etc. [15]. Species distribution models based on indirect signs such as presence/absence or presence-only data have been recently applied to predict the wild boar occurrence from environmental and climatic covariates [6,12,13]. Although habitat suitability models are useful management and conservation tools, their capacity to estimate species abundance is highly controversial as the occupancy may not reflect species density and stability [16,17,18,19,20]. In addition, abundance estimates are needed to study the epidemiology of wildlife diseases, rather than suitability and distribution maps [15]. Wild boar population statistics are mainly available at aggregated spatial level, i.e. by census, hunting or administrative units [7,8,13,15], with little or no information about the distribution of the species within these units. The units can be very heterogeneous in shape, size as well as in terms of land use and environmental characteristics. Aggregated population data does not account for this variation among and within the units [21], and they are difficult to model [22]. Specific disaggregation methods are required to predict population statistics from coarse to fine scale. Recently, multiple linear regression has been used to predict wild boar density at global level [5]. Geostatistics has been successfully used for mapping wild species distribution, including elusive marine mammals, from observations acquired at point or transect level [23], as well as for spatial downscaling and data disaggregation from coarse to fine resolution in land cover mapping [24], human population mapping estimation [21], image sharpening [25], disease mapping [26], and precipitation estimate [27]. Recent studies demonstrated the utility of incorporating the residuals of the regression models to increase interpolation accuracy. In particular, multiple regression and area-to-point kriging of the regression residuals were applied to estimate human population density from coarse census blocks to fine resolution land use maps [21], as well as to downscale precipitation data from coarse to fine resolution rasters [27]. However, to the best of our knowledge, the method has not yet been applied to disaggregate wild boar population data from polygons/units of different shape and size to high resolution raster maps.

We present a geostatistical method based on regression modelling and area-to-point residual kriging to disaggregate wild boar statistics, map and predict its abundance from spatially heterogeneous administrative units (polygons) to high resolution raster maps at 5 km using climatic and environmental covariates. We discuss advantages and limits of the method and its potential application in animal health.

## Materials and methods

### Study area

**Fig 1:**
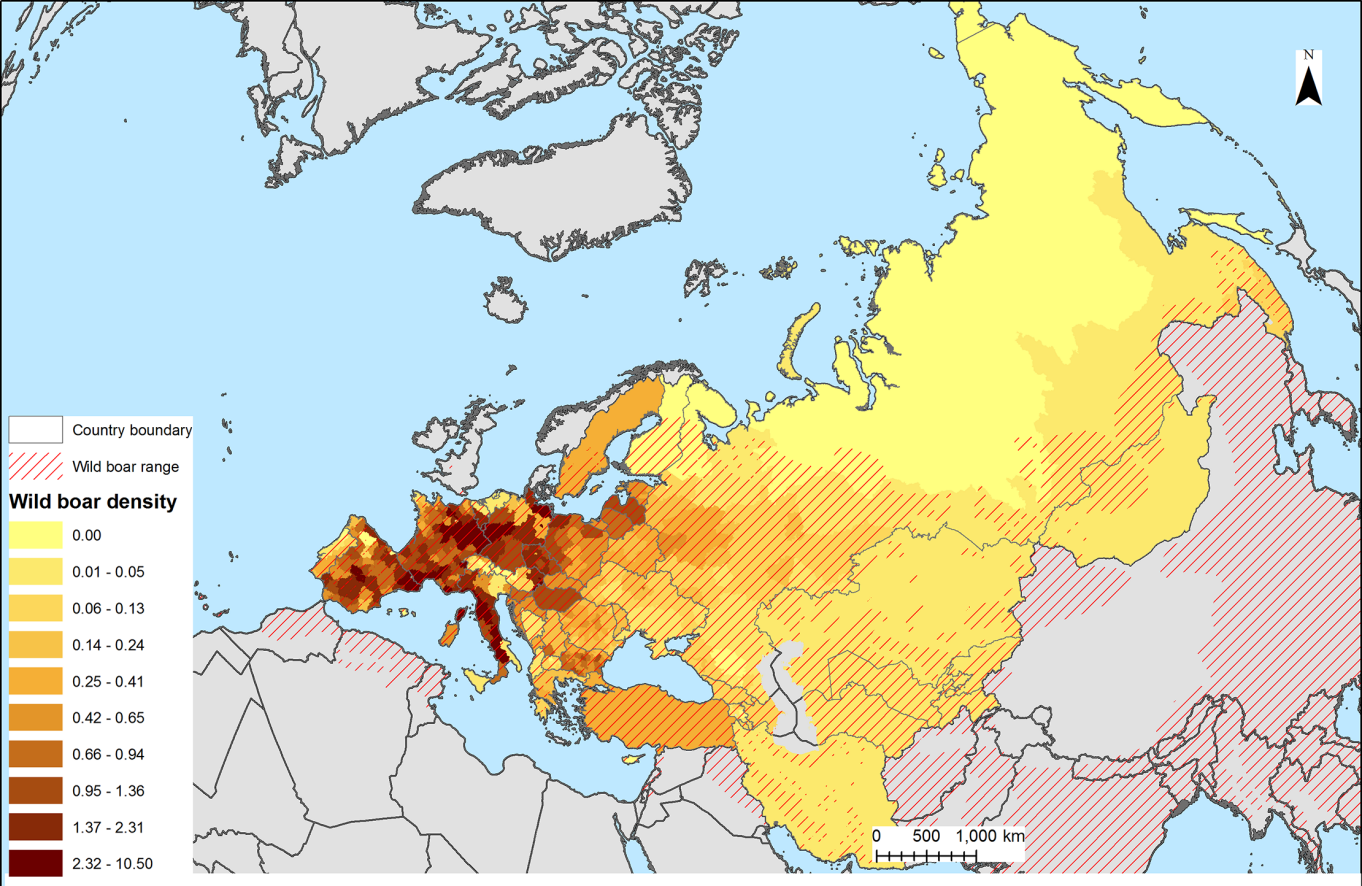
Wild boar density by administrative units (original input data) and wild boar range.

The wild boar population density was estimated from western Europe to central-northern Asia (Fig 1). This area encompasses Mediterranean, Temperate, and Boreal bioclimatic zones [28] with a wide range of vegetation types, climate and elevation [29], which are known to influence the distribution of the species regionally and locally [11].

### Wild boar population data and geographic extent

The wild boar population density was estimated using a dataset available at FAO, including hunting and census data available from different sources and at different level of administrative units [30], for a total of 46 countries and 509 sub-national units (Fig 1 and S1 Table). Censuses and population estimates were acquired for 74% of the countries and were available particularly for eastern Europe, while hunting data were available for 83% of the countries, and mainly for western Europe. Both types of data were acquired for 57% of the countries. This subset was used to calculate the proportion of hunted animals and estimate a correction factor to convert hunting data to densities for those countries presenting hunting data only (n = 8)(S1 Table) [30]. The data were spatially and temporally heterogeneous. National totals were acquired for 27 countries, while sub-national data (level 1 or higher, n = 480) were available for 19 countries. Except for Turkey, Kazakhstan, Turkmenistan, Uzbekistan, the countries with national totals were similar in size to the sub-national units of surrounding countries, thus making the dataset regionally homogeneous and regular in terms of the size and shape of the spatial units. The time frame of the dataset ranged between 1993 and 2011, with 84% of the data acquired after the year 2007. Data earlier than 2000 were obtained from Azerbaijan and Spain (4%). Population, hunting data, correction factors and source data are reported in S1 Table.

The International Union for Conservation of Nature (IUCN) wild boar occurrence map [31] was updated by digitizing detailed maps for Spain, Italy, Iran, Greece and ex-USSR countries found through a literature review [32,33,34,35,36,37] and used to exclude unsuitable wild boar areas inside each administrative unit using a simple GIS overlay operation in ArcGIS (Fig 1). The population density data, expressed as numbers per km^2^ of suitable habitat, were normalized to approximate to a normal distribution using the fourth root power ($x^{\frac{1}{4}}$) transformation prior to perform the statistical analysis [38].

### Predictors

Based on a literature review of environmental and climatic determinants of the wild boar distribution, 18 bioclimatic variables, 3 continuous vegetation cover (i.e. percentage of tree cover, herbaceous vegetation and bare ground) and 2 topographic variables (elevation and slope) were selected as the most significant covariates for predicting wild boar density across the study area [2,5,7,11,12,39,40]. The bioclimatic variables were obtained from the WorldClim online database at 30 arc-seconds (~1 km) spatial resolution for the period 1950–2000 [41]. The vegetation cover grids at 500 m resolution were downloaded from the Global Land Cover Facility [42]. The elevation and slope were derived from the Shuttle Radar Topography Mission (SRTM), 30 arc-second pixel [43]. Average values of the predictors by suitable habitat by wild boar administrative unit were normalized and standardized and used in the statistical analysis. The predictors and properties are listed in Table 1.

**Table 1:** Predictor variables included in the wild boar model-building process.

Variable Name	Description	Resolution	Unit
*Bioclimatic *			
BIO1	Annual Mean Temperature	1 km	Degrees Celsius
BIO2	Mean Diurnal Range (Mean of monthly (max temp—min temp))	1 km	Degrees Celsius
BIO3	Isothermality (BIO2/BIO7) (* 100)	1 km	Percent
BIO4	Temperature Seasonality (standard deviation *100)	1 km	Percent
BIO6	Min Temperature of Coldest Month	1 km	Degrees Celsius
BIO7	Temperature Annual Range (BIO5-BIO6)	1 km	Degrees Celsius
BIO8	Mean Temperature of Wettest Quarter	1 km	Degrees Celsius
BIO9	Mean Temperature of Driest Quarter	1 km	Degrees Celsius
BIO10	Mean Temperature of Warmest Quarter	1 km	Degrees Celsius
BIO11	Mean Temperature of Warmest Quarter	1 km	Degrees Celsius
BIO12	Annual Precipitation	1 km	Millimeter
BIO13	Precipitation of Wettest Month	1 km	Millimeter
BIO14	Precipitation of Driest Month	1 km	Millimeter
BIO15	Precipitation Seasonality (Coefficient of Variation)	1 km	Percent
BIO16	Precipitation of Wettest Quarter	1 km	Millimeter
BIO17	Precipitation of Driest Quarter	1 km	Millimeter
BIO18	Precipitation of Warmest Quarter	1 km	Millimeter
BIO19	Precipitation of Coldest Quarter	1 km	Millimeter
*Vegetation cover *			
Percentage Tree cover	Modis Vegetation continuous fields	500 m	Percent
Percentage herbaceous	Modis Vegetation continuous fields	500 m	Percent
Percentage bare ground	Modis Vegetation continuous fields	500 m	Percent
*Topography *			
Elevation	Elevation	1 km	Meter
Slope	Slope	1 km	Degree

All GIS layers were re-projected to the Polar Lambert Azimuthal Equal Area and re-sampled (with the bilinear interpolation method) to 1 × 1 km^2^ before performing spatial analysis. Spatial analysis was performed in ArcGIS 10.0 (ESRI) and statistical analysis in R 3.1.0 [44].

### Wild boar modelling approach

**Fig 2:**
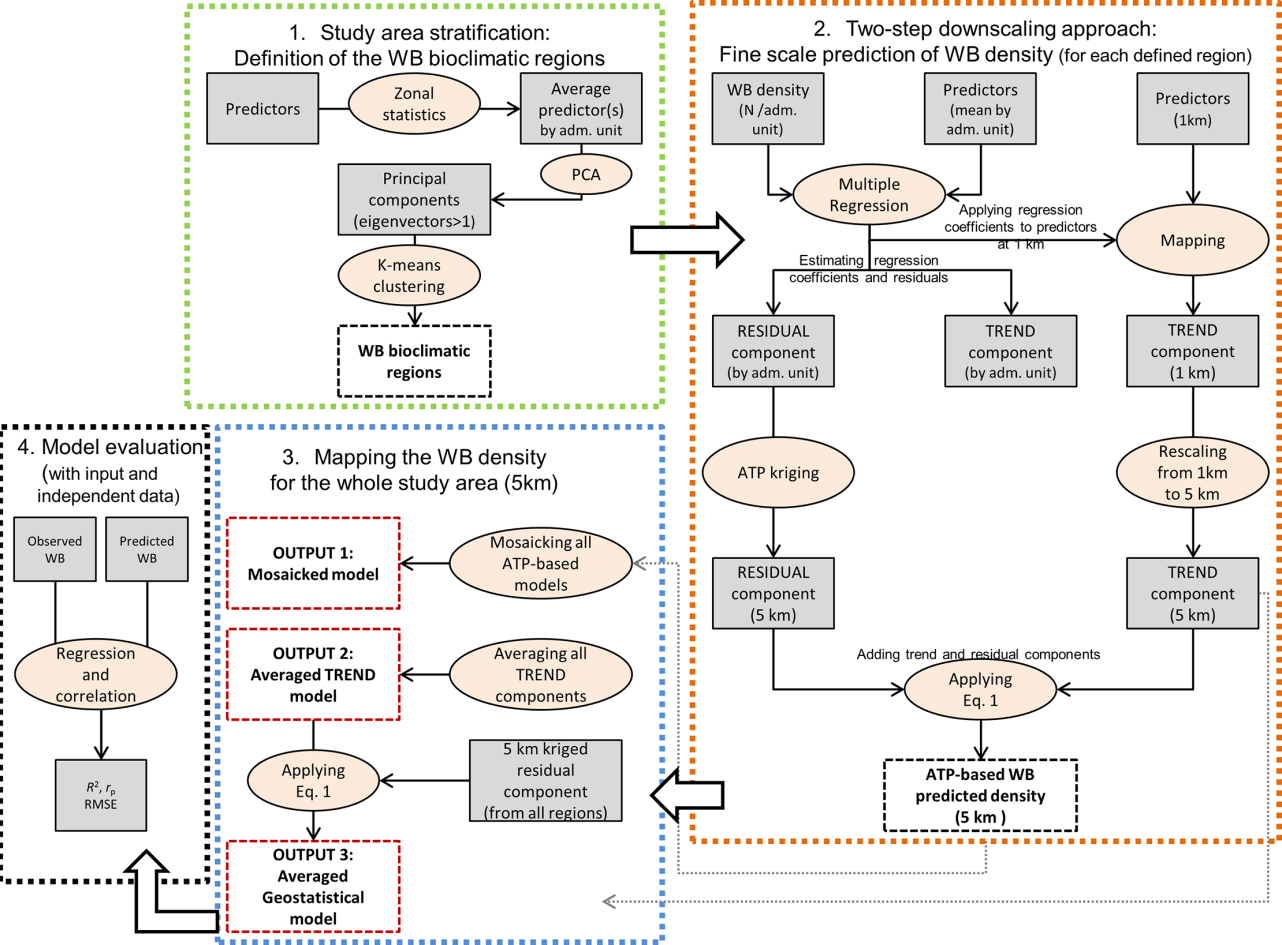
Flowchart illustrating the 4 main steps of the wild boar (WB) density modelling approach.

A flowchart illustrates the four-step modelling approach developed in this study (Fig 2). *First*, we calculated the average value of each predictor by wild boar administrative unit. Given the large geographical and high bioclimatic heterogeneity of the study area, a given predictor variable may be associated quite differently with the species densities in different bioclimatic regions [45]. We therefore applied a stratification method [46] to define regions with similar environmental conditions for the wild boar occurrence and group the wild boar units accordingly. *Second*, for each bioclimatic region, we applied a two-step downscaling geostatistical approach based on multiple regression and area-to-point residual kriging [27] to disaggregate wild boar densities from coarse (polygons) to fine resolution raster maps (5 km). Within the geostatistical framework, the wild boar density was decomposed into trend and residual components. Quantitative relationships between wild boar data and environmental variables by administrative unit were estimated through multiple regression analysis and the coefficients were used to derive the trend components at 1 and 5 km spatial resolution. Then, the residual components i.e., the differences between the trend components and the original wild boar data by administrative units were downscaled at 5 km resolution using area-to-point kriging. The trend and residual components at 5 km resolution were finally added to generate fine scale wild boar estimates for each bioclimatic region. The trend components for each bioclimatic region were also extrapolated to the whole study area. *Third*, we produced three different output maps of predicted wild boar density for the whole study area: (a) a “geostatistical *mosaicked* model” by mosaicking (merging) the predicted wild boar densities obtained for each bioclimatic region; (b) an “averaged *trend* model” by averaging the trend components extrapolated to the whole study area and (c) an “*averaged geostatistical* model” by adding the map to the kriged residual components. *Fourth*, we validated the three final output models using the original wild boar input data as well as an independent dataset. Each step of the process is detailed below. The spatial resolution of 5 km was chosen to match the spatial resolution of livestock distribution maps produced by FAO, including the domestic pig density map [38] to facilitate the analysis of diseases’ transmission among wild and domestic species.

#### Study area stratification: Definition of the wild boar bioclimatic regions

The normalized and standardized average values of the predictors by administrative units were input in a principal component analysis (PCA) to reduce the dimensionality of the environmental dataset into a set of linearly uncorrelated and independent components. The first four principal components (eigenvector >1) were input in the k-means cluster analysis to classify the study area in homogeneous bioclimatic regions. The elbow method was used to define the number of bioclimatic regions [47](Fig 2, box 1).

#### Two-step downscaling approach: Disaggregating and predicting wild boar densities from polygons to 5 km resolution maps

For each bioclimatic region, we applied a two-step downscaling geostatistical approach [27] based on multiple regression and area-to-point residual kriging to disaggregate wild boar population data from coarse scale administrative units (polygons) to fine resolution raster maps (5 km) using auxiliary fine resolution bioclimatic and environmental information. The method decomposed wild boar densities *D*(*u*) in: (a) a deterministic, *trend* component *m*(*u*) (at coarse scale, i.e., polygon level), which indicates the wild boar density (trend) influenced by the bioclimatic and geographic variables, and (b) a stochastic *residual* component *R*(*u*) that accounts for the spatial correlation information of the input wild boar residuals (see Eq 1).

D(u)=m(u)+R(u) (1)

The *trend* component was estimated from the statistical relation between wild boar density and auxiliary environmental variables by administrative units through multiple regression. Under the assumption that attribute values at a coarse scale are linear averages of their constituent fine scale point values, these relationships were applied to the environmental variables in order to estimate the trend component at a fine scale. The stochastic *residual* component was estimated at finer scale by interpolating the regression residuals using the area-to-point kriging [48]. Then the *trend* component was added to the *residual* component to produce a fine resolution wild boar density map (hereafter ATP-based WB predicted density) for each region using the Eq 1. This approach presents the mass preservation property, i.e. it can reproduce the original wild boar density values when the downscaling results at a fine scale are re-aggregated to the coarse scale [27] and it accounts for irregular geographic units (shape, size)[26] (Fig 2, box 2).

#### Theory on regression-based interpolation and area-to-point residual kriging

Consider a study area of *k* wild boar density data by irregular and coarse administrative units {*z*(*v*_*k*_),*k* = 1,…,*K*}, where *v*_*k*_ = *v*(***u***_*k*_) is the *k*th data with its centroid ***u***_*k*_, and *M* auxiliary fine scale environmental variables $\left\{y_{i}^{k}(u_{n}),i=1,\ldots ,M,n=1,\ldots ,N\right\}$ within each *k*th unit. *N* is the number of discretizing points within each unit and its determination depends on the predefined finer scale value. If the environmental variables show linear relationships with the wild boar density values, the latter at both coarse and fine scales can be expressed in terms of the environmental variables via multiple linear regression as:

$$z(v_{k})=a+\sum_{i=1}^{M}b_{i}\;y_{i}(v_{k})+R(v_{k}),$$ (2)

$$z^{k}(u_{n})=a+\sum_{i=1}^{M}b_{i}\;y_{i}^{k}(u_{n})+R^{k}(u_{n}),$$

where *a* and *b*_*i*_ are regression coefficients for the intercept and slope of the *i*th variable, respectively. *z*^*k*^(***u***_*n*_) is the downscaled wild boar value at a target finer scale within the *k*th unit. *R*(*v*_*k*_) and *R*(***u***_*n*_) are the residual components at coarse and fine scales, respectively, which cannot be accounted for by environmental variables. If the original wild boar data, environmental variables, and the residual components at a coarse scale can be expressed by the average values of *N* fine scale data within each unit, respectively, Eq (2) can be reformulated as (3)

$${z(v}_{k})=a+\sum_{i=1}^{M}b_{i}[\frac{1}{N}\sum_{n=1}^{N}y_{i}^{k}\left(u_{k}\right)]+\frac{1}{N}\sum_{n=1}^{N}R^{k}(u_{n})$$ (3)

$${z(v}_{k})=\frac{1}{N}\sum_{n=1}^{N}\left[a+\sum_{i=1}^{M}b_{i}y_{i}^{k}(u_{n})+R^{k}(u_{n})\right]$$

$${z(v}_{k})=\frac{1}{N}\sum_{n=1}^{N}z^{k}(u_{n})$$

The trend component at a finer scale was estimated by applying the regression relationships obtained at course scale to the environmental variables. The residual component at finer scale was predicted using area-to-point simple kriging [27] of the residual components available at a coarse scale. Area-to-point simple kriging predicts the residual component values at a fine scale by a linear combination of neighboring attribute values at a coarse scale [26,27]. Briefly, variogram deconvolution was applied to the regression residuals for each region to estimate the unknown point-support variogram of the residuals.

A detailed explanation of the area-to-point kriging can be found elsewhere [21,26,27]. Area-to point residual kriging was implemented using SpaceStat 2.0 (BioMedware).

The normalized and standardized predictors were tested for multicollinearity prior to performing the multiple regression because multicollinearity may violate statistical assumptions and may alter model predictions [49]. Different methods exist to evaluate multicollinearity in regression analysis, including correlation matrix, PCA, Tolerance and Variance Inflation factor [50]. In this study we chose the VIF. Predictors with VIF larger than 2.5 were excluded from the multiple regression [51,52]. The variables were tested one by one. A (forwards) stepwise variable selection procedure was applied to select a parsimonious set of predictors for the multiple regression modelling. The method is explained in detail elsewhere [50,51,52].

#### Mapping the wild boar predicted densities for the whole study area (5 km)

The first output (“geostatistical *mosaicked* model”, hereafter mosaicked model) was obtained by mosaicking the ATP-based wild boar density maps predicted for each bioclimatic region in a single, seamless map for the study area (see Fig 2, box 3). In order to minimize the abrupt changes along the boundaries of adjacent regions, a blending image processing technique was applied to the input maps. Specifically, a buffer of 100 km was built around the boundary of each region and used as a mask to average inside the wild boar predicted densities obtained from models of adjacent regions.

Two additional modelling outputs were generated and compared with the mosaicked model: 1) the averaged *trend* model (output 2, Fig 2, box 3), which was derived by averaging the trend components of each bioclimatic region extrapolated at 1 km resolution to the whole study area and resampled at 5 km resolution; 2) the averaged *geostatistical* model (output 3, Fig 2, box 3), obtained by adding the kriged residual component to the averaged *trend* model. The residual component was generated from an ordinary kriging of all regression residuals.

#### Model evaluation and accuracy assessment

The predictive performance of the models was assessed using the wild boar densities of the original input data as well as an independent dataset (Fig 2 box 4). Specifically, the overall accuracy of the averaged *trend* model, averaged *geostatistical* model and the *mosaicked* model were measured by calculating the averaged predicted densities by administrative unit and then relating these averaged predicted density to the averaged observed density of the input data. Given the mass preservation of the ATP-based method [26], a 1:1 relationship was expected. Model performance was also evaluated for each region. In addition, the accuracy across the whole wild boar study area was independently assessed using the density data reported by Melis *et al*. [11] for 54 locations in Eurasia. These locations were georeferenced and overlaid with the three output models. The predicted wild boar densities extracted at those locations were therefore related to the densities observed by Melis *et al*. [11].

The Pearson correlation coefficient (*r*_*p*_), coefficient of determination (*R*^*2*^) and root mean square error (RMSE) calculated between predicted and observed densities were used to assess model accuracy [53].

## Results

**Fig 3:**
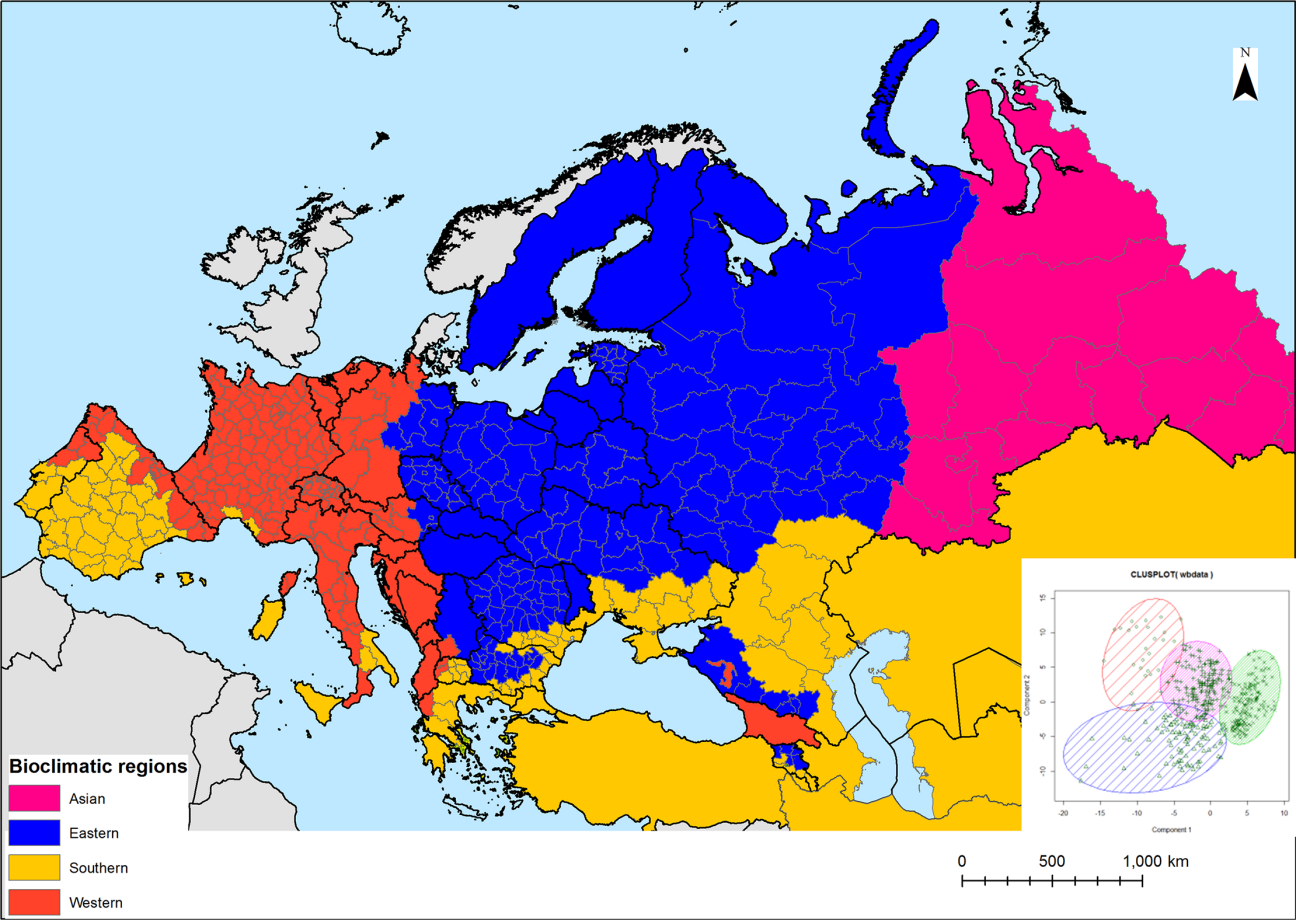
Bioclimatic regions for the wild boar as defined by PCA and cluster analysis. The cluster plot of the first and second components is shown in the inset. The symbols represent the administrative units grouped in the 4 clusters/regions: Asian (red), eastern (pink), western (green) and southern (blue).

The k-means cluster analysis grouped the wild boar units into four main homogeneous bioclimatic regions for the wild boar occurrence: 1) Asian, 2) eastern; 3) western, and 4) southern (Fig 3). The units within each region were similar in size and shape, except for southern, which included the largest units (Iran, Turkey, Kazakhstan, Uzbekistan and Turkmenistan).

Elevation and slope were highly correlated with most of the bioclimatic variables (Pearson correlation coefficient, *p* <0.05) and were therefore excluded from the geostatistical modelling. The results of multiple regression models performed for each bioclimatic region are reported in Table 2. The variance explained by the trend components ranged between 49 and 53%, indicating that regression alone is not sufficient to predict the wild boar density in each region. The predictors were reduced to 2–6 significant variables and differed among regions. In the Asian region (pink, Fig 3) wild boar density was positively related to annual mean temperature (BIO1) and negatively related to both precipitation of coldest quarter (BIO19) and continuous herbaceous cover. Instead, in the eastern region (blue) wild boar density was negatively related to both temperature annual range (BIO7) and precipitation of wettest month (BIO13), but positively associated with mean temperature of wettest quarter (BIO8) and continuous tree cover. In the southern region (yellow), a significant positive association was found with mean diurnal range (BIO2), minimum temperature of coldest month (BIO6), and continuous tree cover. In the western region (orange) wild boar density was positively related to annual mean temperature (BIO1) and continuous tree cover, but negatively related to mean diurnal range (BIO2), mean temperature of wettest quarter (BIO8), precipitation seasonality (BIO15) and precipitation of coldest quarter (BIO19). Given the lower variance explained by the regression model for the Asian region, as well as the large residuals associated with the largest units in the southern region (i.e. Iran, Kazakhstan, Uzbekistan and Turkmenistan), the Asian model was excluded from the geostatistical modelling and the boundary of the southern region was delimited along by the Ural mountains and the Caspian sea.

**Table 2:** Results of the multiple regression models by bioclimatic region: Standardized coefficients and standard errors (in brackets), adjusted R^2^, sample size (N), F value and degrees of freedom (dfs), residual standard error (RSE) and *p*-values.

Predictors	Asian	Eastern	Southern	Western
(Intercept)	1.21	7.19	-0.87	2.13
Annual Mean Temperature (BIO1)	0.04			0.08
Mean Diurnal Range (BIO2)			1.45	-1.11
Min Temperature of Coldest Month (BIO6)			0.03	
Temperature Annual Range (BIO7)		-3.60		
Mean Temperature of Wettest Quarter (BIO8)		0.02		-0.01
Precipitation of Wettest Month (BIO13)		-0.92		
Precipitation Seasonality (BIO15)				-0.61
Precipitation of Coldest Quarter (BIO19)	-0.05			-0.07
Continuous herbaceous cover	-0.04			
Continuous tree cover		0.25	0.28	0.80
R^2^ adjusted	0.51	0.53	0.50	0.49
*N*	26	176	94	171
*F*	9.67	50.2	31.5	28.5
*dfs*	(3)(22)	(4)(171)	(3)(90)	(6)(164)
*RSE*	0.07	0.17	0.23	0.22
*p*	<0.05	<0.05	<0.05	<0.05

**Fig 4:**
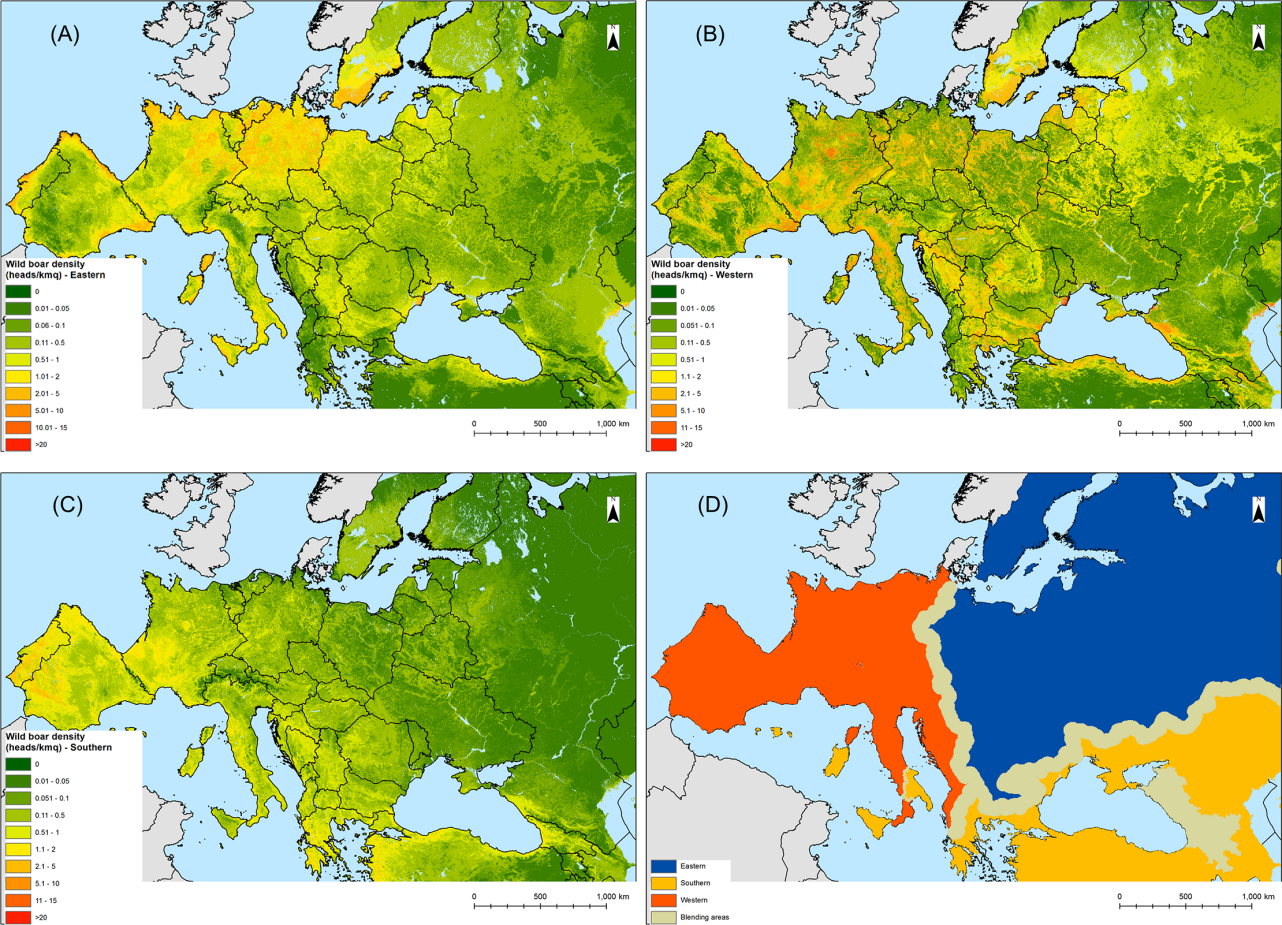
Trend components derived from the regression relationship obtained for (A) the eastern, (B) western and (C) southern regions and extrapolated to the whole study area respectively. (D) Redefined bioclimatic regions and blending zones. The Asian region is not shown as it was excluded from the geostatistical analysis.

**Fig 5:**
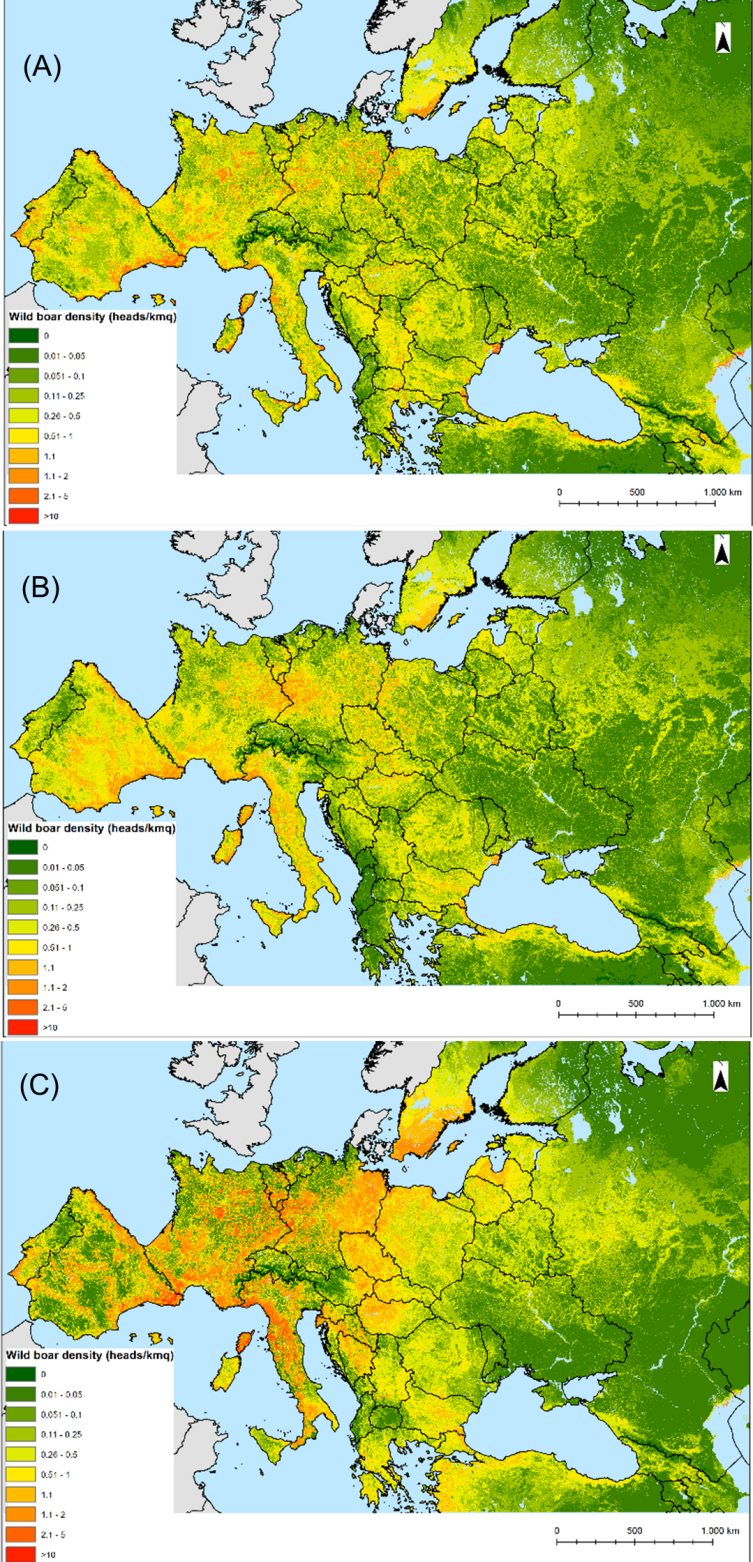
**Model outputs: average trend (A), average geostatistical model (B) and mosaicked model (C)**.

The trend components generated by applying the three regression relationships to the environmental variables at 1 km resolution and extrapolated for the whole study area at 5 km resolution are displayed in Fig 4A–4C. The results of the variogram deconvolution for each region are shown in S2 Table, while an example of the area-to-point residual kriging is given for the eastern region in S1 Fig. The predicted wild boar population-densities by region obtained by adding trend and residual components are shown in S2 Fig. When the downscaled results at 5 km were re-aggregated at the original administrative unit level, the preservation of the coherence (mass) property was highest for the eastern region (Pearson correlation coefficient *r*_p_ = 0.7), western (*r*_p_ = 0.6) and lowest for the southern region (*r*_p_ = 0.4). The wild boar densities predicted in the Iberian Peninsula (southern region) were less accurate than those obtained using the regression coefficient of the western region. Consequently, the western region was modified to include the Iberian Peninsula and the mosaic mask for generating the final prediction map was adjusted accordingly (Fig 4C). The final wild boar population-density models, i.e. averaged trend, average geostatistical and mosaicked models, are shown in Fig 5A–5C. Since population density cannot be less than 0, a small proportion of negative estimates (1%), mainly located in mountainous areas (i.e., Pyrenees, Alps, Caucasus) was adjusted to 0.

The model performance assessed using the wild boar input data increased substantially from the average trend model (t = 17.38, df = 456, *p*<0.05, Pearson correlation coefficient *r*_p_ = 0.63), to averaged geostatistical model (t = 24.5, df = 456, *p*<0.05, *r*_p_ = 0.75), and it was highest for the mosaicked model (t = 32.1, df = 456, *p*<0.05, *r*_p_ = 0.83), though a 1:1 relation was not found. We found a significant positive correlation between the mosaicked model wild boar predicted densities and the densities reported by Melis *et al*. [11] (t = 9.16, df = 43, p<0.001; *r*_p_ = 0.81).

Table 3 shows the *r*, MAE and RMSE of the validation analysis for the three models with input and independent data.

**Table 3:** Validation results.

Model name	*r*_*p*_	adjusted *R*^2^	RMSE	*p*
Output 1: Mosaicked model	0.83	0.69	0.17	<0.001
Output 2: Averaged Trend model	0.63	0.40	0.13	<0.001
Output 3: Averaged Geostatistical model	0.75	0.57	0.21	<0.001

## Discussion

Wild boar population statistics are mainly available at aggregated level, or as scattered observations, particularly for large geographical areas [5,7]. However, the management and control of wild boar populations require accurate and detailed spatial information on species distribution and abundance. Two recent studies have extrapolated and predicted wild boar distribution and expansion at global level using multiple linear regression [5] and Bayesian approaches [6] based on wild boar data available across parts of geographical range of the species. In our study, a multi-step geostatistical approach was proposed, tested and validated to disaggregate wild boar population density statistics from polygons of irregular size and shape to fine spatial resolution maps. The approach represents a valuable downscaling tool that could be applied to any population statistics, including other wildlife species, livestock, human, as well as disease data. Strengths and limitations of the approach are discussed below.

The first strength of the approach is the stratification step. Differently from other studies [5,6,7,8], our novel method was able to account for the large geographical and high bioclimatic heterogeneity of the study area and to define four different biogeographic/bioclimatic regions—Asian, southern, eastern, and western—reflecting different spatiotemporal patterns of food resources, shelter/cover, available to the species. By analyzing each region separately, a parsimonious set of specific predictors and limiting factors for wild boar distribution and density was identified for each region: winter harshness in the Asian and eastern regions (as indicated by the negative relation with Precipitation of Wettest Month (BIO13) and Precipitation of Coldest Quarter (BIO19) respectively); forest productivity and shelter in western, eastern and southern regions (as indicated by the positive relation with tree vegetation cover); extreme temperature and precipitation variability (e.g., droughts, floods) in western region (as indicated by the negative relation with Precipitation seasonality (BIO15) and Annual Mean Diurnal Range (BIO2)); low habitat heterogeneity and lack of shelter in the Asian region (as indicated by the negative relation with the herbaceous vegetation cover). These results confirmed that winter severity, temperature and precipitation anomalies, as well as vegetation structure, are main macroecological determinants of the wild boar distribution and abundance in northern and temperate latitudes, respectively [5,7,11,12,14], as they affect population dynamics, particularly the survival of newborn piglets. Differently from previous studies [12,39], slope and elevation were not significant predictors, suggesting that terrain is an important species determinant at local scale but not at regional scale.

The main advantage of the approach is the geostatistical framework (step 2), which decomposed the wild boar density in *trend* and *residual* components. Under this framework, the quantitative relationship found between wild boar densities and environmental variables at coarse scale (administrative units) through the regression analysis was used to derive the trend component at finer scale (1km and 5 km). The low accuracy of the trend models was in agreement with the results obtained by other studies [21,27,54], indicating that regression alone cannot account for the within-units environmental heterogeneity and its influence on species distribution and abundance.

The accuracy of the regression-based trend models varied among the regions: the western and the eastern regions showed the lowest (*R*^2^ = 0.49) and highest (*R*^2^ = 0.53) accuracy respectively. The wild boar density in the western region was mainly estimated from hunting data, while the southern region was mainly characterized by larger and irregular units, suggesting that low quality of the input data (hunting *vs*. census, [4]), differences in years covered by the data series [11], as well as units variability in size and shape can impact model performance.

As expected, the predictive performance of the models increased with the incorporation of the regression residuals through the area-to-point kriging. This result was in line with the finding of other studies [21,27] due to the ability of the Area-to-point kriging to account for the stochastic component and the spatial autocorrelation of the aggregated input data. The accuracy was highest for the mosaicked model (*r* = 0.83), and lowest for the averaged trend model (*r* = 0.63), meaning that the statistical relations between wild boar abundance and bioclimatic variables cannot be extrapolated to areas outside the training regions [55]. This is clearly shown by the trend maps (Fig 4A–4C), which display different distribution patterns of wild boar among the bioclimatic regions and low predictability outside the training regions. This finding suggests that model transferability remains an issue in species distribution models [56]. This result further corroborates the importance of defining bioclimatic regions based on statistical stratification methods. However, high environmental variability in certain areas, as well as anthropogenic factors, may also impact the accuracy of the models [5]. In our study, the model performance for Spain based on the regression coefficients of the southern region was low, but improved when we applied the coefficient of the adjacent western region. The two model outputs were discussed with wild boar experts in Spain, who provided a visual validation of the two maps, confirming the higher accuracy of the western model. This result was explained by the high environmental and topographic heterogeneity of the country [12] as well as the wide use of supplementary feeding in some areas [45], which could not be captured by the predictors of the southern region. High environmental heterogeneity was a positive predictor for the wild boar distribution and range expansion in recent studies [5,6,8].

Although we found a strong relation between predicted and observed wild boar densities (*r* = 0.83), the expected 1:1 relation was not found. As highlighted by Liu *et al*. [21], the mass-preserving property of the area-to-point kriging is lost when negative estimates are reset to 0.

The model accuracy was very high even when assessed against independent wild boar data [11]. The biogeographical West-East gradient in wild boar density observed by Melis *et al*. [11] was also found in our model. This longitudinal decline in wild boar densities is mainly related to milder climatic conditions as well as higher vegetation productivity and biodiversity (i.e., tree species with edible seeds for wild boar) in western and southern Europe as compared to the eastern Eurasian range [11].

In synthesis, the approach optimized the use of available coarse resolution abundance data for producing high resolution density maps. Although the geostatistical method accounted for units of different size and shape, the quality of the input data and high variability in size and shape impacted model performance. In this study the approach was tested using main climatic and environmental predictors for the wild boar. Additional predictors such as metrics of landscape fragmentation [32] could increase model performance [8]. The application of Poisson regression could be tested to improve the regression-based trend interpolation [57]. In addition to the mean value, other measures of the variability of the predictors by administrative unit (e.g., range, coefficient of variation) could be explored to improve model prediction, particularly for regions with high environmental heterogeneity. Given the availability of new areas, more recent or more precise wild boar and geographic data, the model can be easily updated, as well as applied to other geographic areas where the species occurrence is expanding (e.g., China, United States of America).

Wild boar distribution and density maps can become useful tools to assess the growing threat that these populations pose to agriculture (i.e. crop damage), conservation, road traffic, and health (livestock, wildlife, and even human). Such maps will allow to first assess the situation, and then implement management actions accordingly, in an attempt to solve, or at least minimize, the negative effects. The next section expands on how such assessment and management applies specifically to animal health, which is a topic of particular urgency and concern given the current progressive spread of ASF in Europe, which often involves wild boar. An important objective of the FAO is to disseminate the data. The predicted wild boar densities based on the mosaicked model are freely available and can be downloaded from S1 Geodataset of the Supporting Information, provided the original authors and source are credited.

### Applications to animal health management

Wildlife and livestock connect through different paths, which allows for disease transmission in both directions. This has clear implications in health management, as the objective is to keep livestock and wildlife healthy, by preventing the introduction of diseases from one population to another. Sometimes, there is also a public health component/concern. Wild boar distribution and density maps can be an extremely useful tool for veterinary services, wildlife managers and epidemiologists to prevent and control animal diseases. When incorporated into risk analysis or disease risk modelling, such maps allow the identification and assessment of the specific pathways and risks posed by wild boar in the introduction, spread and maintenance of animal diseases in a certain region, and their potential subsequent spread to livestock (usually domestic pigs) and *vice versa*. In this line, spatial models have been recently developed to simulate the introduction and spread of classic swine fever and foot-and-mouth disease in wild pigs in Australia, allowing for the testing of the effectiveness of different control measures and surveillance strategies [58,59,60]. Similar models based on accurate maps of wild boar densities will allow identifying the potential corridors of introduction, the areas of highest densities, or where wild boar-domestic pig interactions are more likely (e.g. where backyard or free-ranging pigs, and other low biosecurity production systems exist; unsecured dumping sites which may contain infected pig products; or through hunters). As a result, early detection strategies in high-risk areas can be planned and implemented, e.g. strengthening passive surveillance, testing hunted animals or road kills, or through targeted surveillance (non-invasive sampling or capture and release). Prevention measures can also be applied in high risk areas. Those applied to the domestic pig sector will be most effective in preventing infection in both directions, e.g. double-fencing, permanent enclosure of animals, proper disposal of kitchen and slaughtering waste, and other biosecurity improvements. Although controversial and still subjected to extensive debate, there are intervention measures to prevent, or at least minimize, the entry of infected wild boar into certain areas (repellents, fences, hunting pressure, etc.). There are also management options if a disease becomes established in wild boar: vaccination, carcass removal, ban of supplementary feeding, or hunting strategies.

The precise set of measures will depend to a certain extent on the mechanism of transmission of the disease, but in all the cases, knowing the numbers and distribution of wild boar will greatly help to plan the strategies and estimate the efforts/resources needed.

## Supporting information

S1 Fig ATP residual kriging for the eastern region. (TIF)

S2 Fig **Predicted wild boar density by region based on ATP-regression models**: (A) eastern, (B) southern, (C) western, and (D) original input density data by administrative unit. Legend: Low (green)–High (red) density values.
(TIF)

S1 Table Wild boar source data. (DOCX)

S2 Table Results of the variogram deconvolution for the three bioclimatic regions and relative charts. (DOCX)

S1 Geodataset Predicted wild boar densities based on the mosaicked model. (ZIP)
