# Supplementary figures and images for: Wild boar mapping using population-density statistics: From polygons to high resolution raster maps
Source: PLoS One. 2018 May 16;13(5):e0193295. doi: 10.1371/journal.pone.0193295 (PMC5955487; doi:10.1371/journal.pone.0193295)

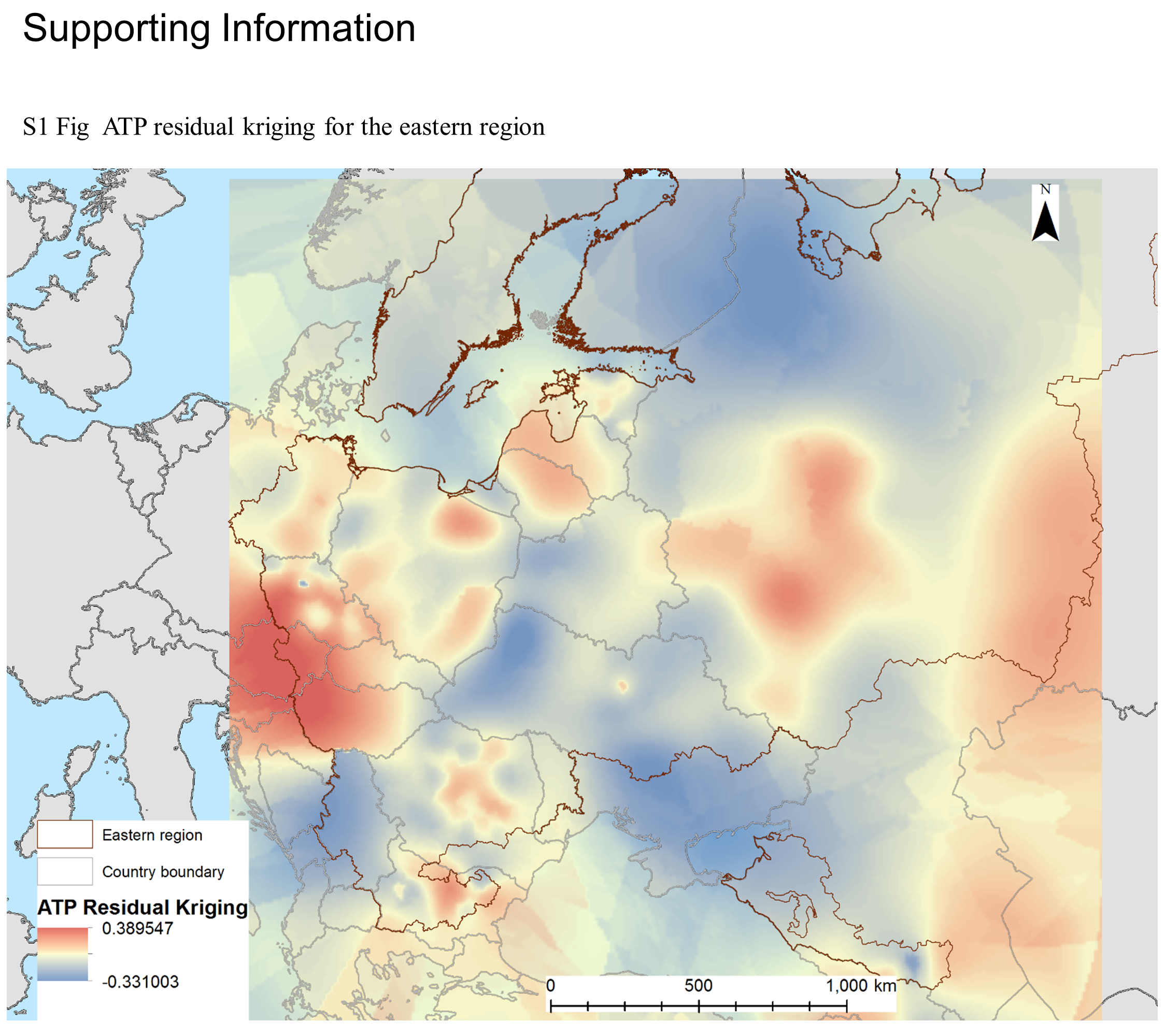

Supplement: S1 Fig — (TIF) [file pone.0193295.s001.tif]

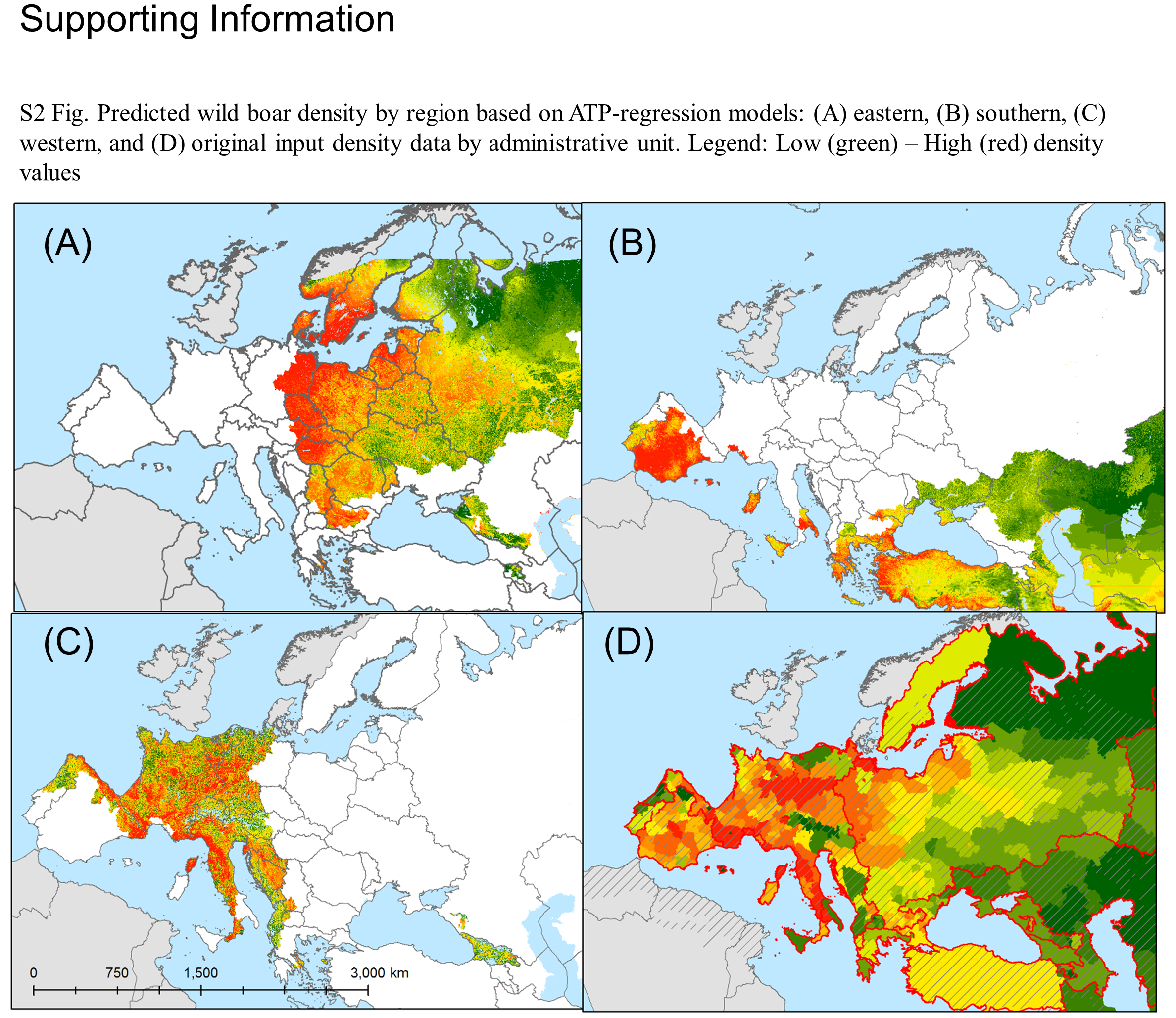

Supplement: S2 Fig — Predicted wild boar density by region based on ATP-regression models: (A) eastern, (B) southern, (C) western, and (D) original input density data by administrative unit. Legend: Low (green)–High (red) density values. (TIF) [file pone.0193295.s002.tif]
