# Supplementary material for: Wild boar mapping using population-density statistics: From polygons to high resolution raster maps
Source: PLoS One. 2018 May 16;13(5):e0193295. doi: 10.1371/journal.pone.0193295 (PMC5955487; doi:10.1371/journal.pone.0193295)
Supplement: S1 Table — (DOCX) [file pone.0193295.s003.docx]

**Supporting Information**

The wild boar data were collected, collated and estimated from a variety of sources by a previous FAO project to investigate African swine fever (ASF) introduction and spread in Eurasia**^[[1]](#endnote-1)^**^, ii, iii, iv^.

**S1 Table Wild boar source data**

| Reference | COUNTRY | Data | Year/s of estimate | Units (n) | Population (counted or estimated) | Harvest (actual or estimated) | Correction factor (F) |
| --- | --- | --- | --- | --- | --- | --- | --- |
| 1 | Albania | Population | 2000 | 1 | 1000 | 636 | 0.63 |
| 2 | Andorra | No Data | - | 1 | - | - |  |
| 3 | Armenia | Population | 2009 | 10 | 1080 | 686 | 0.63 |
| 4 | Austria | Harvest | 2009 (2005) | 9 | 60000 | 30212 | 1.27 |
| 5 | Azerbaijan | Population | 1993 | 1 | 7500 | 4766 | 0.63 |
| 6 | Belarus | Both | 2008 (2010) | 6 | 69100 | 25949 | 1.23 |
| 7 | Belgium | Both | 2008 - 2011 | 2 | 26203 | 22307 |  |
| 8 | Bosnia & Herzegovina | Harvest | 2009 | 1 | 2212 | 1406 | 1.27 |
| 9 | Bulgaria | Both | 2010 | 28 | 68903 | 20851 |  |
| 10 | Croatia | Harvest | 2009 | 1 | 28707 | 18243 | 1.27 |
| 11 | Cyprus | Both | 2006 | 1 | 0 | 0 |  |
| 12 | Czech Republic | Both | 2010 | 14 | 121690 | 57880 |  |
| 13 | Estonia | Both | 2010 | 15 | 22650 | 17028 |  |
| 14 | Finland | Both | 2009 | 1 | 400 | 110 |  |
| 15 | France | Harvest | 2009 | 96 | 1000000 | 501484 | 1.99 |
| 16 | Georgia | Both | 2000 | 1 | 5000 | 1000 |  |
| 17 | Germany | Harvest | 2010 | 16 | 1000000 | 340706 | 2.94 |
| 18 | Greece | Population | 2009 | 6 | 19033 | 12095 | 0.63 |
| 19 | Hungary | Both | 2009 | 1 | 106700 | 111200 |  |
| 20 | Iran | Harvest | 2009 | 1 | ??? | 2500 |  |
| 21 | Italy | Harvest | 1999 - (2005) | 20 | 600000 | 159714 | 3.76 |
| 22 | Kazakhstan | Both | 2008 | 1 | 17564 | 1132 |  |
| 23 | Kosovo | Population | 2008 | 1 | 10000 | 6355 | 0.63 |
| 24 | Kyrgyzstan | Both | 2000 | 1 | 8000 | 800 |  |
| 25 | Latvia | Both | 2010 | 1 | 67200 | 30201 |  |
| 26 | Liechtenstein | No Data | - | 1 | - | - |  |
| 27 | Lithuania | Both | 2009 | 1 | 54608 | 41441 |  |
| 28 | Luxembourg | Harvest | 2008 | 1 | 8655 | 5500 | 1.27 |
| 29 | Macedonia | Both | 2009 | 8 | 1889 | 490 |  |
| 30 | Moldova | Both | 2000 | 1 | 5000 | 1000 |  |
| 31 | Mongolia | Both | 2005 | 1 | 50000 | 30000 |  |
| 32 | Montenegro | Both | 2009 | 1 | 3584 | 482 |  |
| 33 | Netherlands | Population | 2011 | 1 | 860 | 547 | 0.63 |
| 34 | Poland | Both | 2009 | 16 | 227900 | 197977 |  |
| 35 | Portugal | Harvest | 2000 | 7 | 13847 | 8800 | 1.27 |
| 36 | Romania | Both | 2009 | 41 | 58988 | 13787 |  |
| 37 | Russian Federation | Both | 2010 | 85 | 404570 | 63957 |  |
| 38 | Serbia | Both | 2009 | 1 | 17475 | 5811 |  |
| 39 | Slovakia | Both | 2009 | 1 | 31652 | 31473 |  |
| 40 | Slovenia | Harvest | 2008 | 1 | 14370 | 9132 | 1.27 |
| 41 | Spain | Harvest | 1999 - 2008 (2005) | 48 | 600000 | 153596 | 3.91 |
| 42 | Sweden | Both | 2010 | 1 | 150000 | 65000 |  |
| 43 | Switzerland | Harvest | 2009 | 26 | 6501 | 4132 | 1.27 |
| 44 | Tajikistan | Both | 2000 | 1 | 4000 | 257 |  |
| 45 | Turkey | Population | 2010 | 1 | 275000 | 1060 (173250) | 0.63 |
| 46 | Turkmenistan | Both | 2000 | 1 | 4000 | 600 |  |
| 47 | Ukraine | Both | 2007 | 27 | 48982 | 4547 |  |
| 48 | Uzbekistan | Both | 2000 | 1 | 4000 | 200 |  |
|  |  |  |  |  |  |  |  |

**Reference list**

(1) Bego F, Peja N, Pllaha S (2004) Large Carnivores (Bear, Lynx and Wolf) in Albania. Unpublished Report, Albanian Society for the Protection of Birds and Mammals (ASPBM) and Transborder Wildlife: 6.

(3) Armenia FAO field Officer (Official Census Data)

(4) Weidwerk, Jagdstatistik in Österreich (2009) <http://www.weidwerk.at/html/stat_2009.htm>

(5) Мусаев МА р (2000) Животный мир Азербайджана. Позвоночные. Баку, Элм. C. 594-596

(6) Алехнович АВ, Бычков ВП, Востоков ЕК, Лях ЮГ, Ризевский ВК, Шакун ВВ (2011) Глава 8. Животный мир. "Состояние природной среды Беларуси" (экологический бюллетень 2010 г.). В.Ф.Логинов (ред.).НАЦИОНАЛЬНАЯ АКАДЕМИЯ НАУК БЕЛАРУСИ, МИНИСТЕРСТВО ПРИРОДНЫХ РЕСУРСОВ, И ОХРАНЫ ОКРУЖАЮЩЕЙ СРЕДЫ, Минск, 2011. <http://www.minpriroda.gov.by/ru/bulleten/new_url_2108832364>

(7) Bellayachi, el al. (2010). Tableau de bord de l’environnement wallon. Rapportsurl’état del’ environnement wallon V. BRAHY. Jambes, Service Public de Wallonie. Direction générale opérationnelle de l’Agriculture, des Ressources naturelles et de l’Environnement: 232.

(7) Walton D (2011) The return of the boar. Flanders Today, 9 February 2011. <http://freelancejournalism.files.wordpress.com/2011/04/wild-boar.pdf>

(8) Statistical Yearbook (2010) Federation of Bosnia and Herzegovina. <http://www.fzs.ba/Godisnjak2010.pdf>

(9) Bulgaria (2010) MoA Bulgaria, FAO field officer, Official census data 2010

(10) Croatian Bureau of Statistics (2011) <http://www.dzs.hr>

(11) Hadjisterkotis EP, Heise-Pavlov M (2006) The failure of the introduction of wild boar *Sus scrofa* in the island of Cyprus: a case study. Eur J Wildl Res 52: 213–215 DOI 10.1007/s10344-006-0037-3

(12) Czech Statistical Office (2011) <http://www.czso.cz>

(13) Statistics Estonia (2011) http://www.stat.ee

(14) FGFRI (2011) Finnish Game and Fisheries Research Institute. Annual game bag. (<http://www.rktl.fi/english/statistics/hunting/>)

(14) EVIRA, Oravainen J, Sahlström L, Lyytikäinen T (2011) Possible routes of entry into the country for African swine fever - Risk profile. Evira Research Reports 5/2011. <http://www.evira.fi/portal/en/evira/publications/?a=view&productId=257>

(15) ONCFS-FNC-FDC (2011), Réseau ongulés sauvages, Les tableaux de chasse ongulés sauvages pour la saison saison 2008 – 2009 sont disponibles http://www.oncfs.gouv.fr

(16, 24, 30, 44, 46, 48) Danilkin AA (2002) Suids (Suidае). Mammals of Russia and adjacent areas. M.: GEOS, 2002. – 309 p. (In Russian)

(17) DJV (2012) Weitere Statistiken unter www.jagdnetz.de Jahresstrecke Schwarzwild Deutscher Jagdschutzverband, Handbuch 2012. http://medienjagd.test.newsroom.de/201011_strecke_schwarzwild2.pdf

(18) Tsachalidis EP, Hadjisterkotis E (2009) Current distribution and population status of wild boar (Sus scrofa L.) in Greece. Acta Silv. Lign. Hung., Vol. 5: 153-157

(19) Hungarian Central Statistical Office (2010) <http://www.ksh.hu>

(20) Karami M, Mhammadi H, Cheraghi S (2010) Present distribution, abundance, and problems of wild pig (Sus scrofa) in Iran. Wild Pig Conference: science and management. April 11-13, 2010. Crowne Plaza Grand Hotel, Pensacola, Florida, USA. <http://www.wildpigconference.com/proceedings09/karami.pdf>

(21) Carnevali L, Pedrotti L, Riga F, Toso S (2009) Banca Dati Ungulati:Status, distribuzione, consistenza, gestione e prelievo venatorio delle popolazioni di Ungulatiin Italia. Rapporto 2001-2005. Biol. Cons. Fauna, 117:1-168 [Italian-English text]

(22) Охрана окружающей среды и устойчивое развитие» Казахстана. Статистический сборник./Под редакцией А. Смаилов / - Астана, 2009, - 134 стр.

(23) Anonymous (2009) European Commission. DG for Health and Consumers.Transboundary co-operation on CSF eradication control and monitoring between the EU and neighboring countries. PPT presentation. Available on-line at <http://www.oie.int/RR-Europe/eng/Projects/Gftads10-SANCO-CSF.pdf>

(25) Statistics Latvia, 2010 (<http://www.csb.gov.lv>)

(27) Statistics Lithuania, 2009 (<http://www.stat.gov.lt>)

(28) Bulletin technique de l'Administration de la nature et des forêts en matière de gestion de la faune sauvage et de chasse, no. 1 (2010) 36 pp. <http://www.environnement.public.lu/chasse/publications/bulletintechnique1/BT1.pdf>

(29) Forestry: 2009. Skopje: State Statistical Office of the Republic of Macedonia, 2009. – 38 p.

Statistical review / State Statistical Office of the Republic of Macedonia. Agriculture. <http://www.stat.gov.mk>

(31) Wingard JR, Zahler, P (2006) Silent Steppe: The Illegal Wildlife Trade Crisis in Mongolia. Mongolia Discussion Papers, East Asia and Pacific Environment and Social Development Department. Washington D.C.: World Bank.

(32) Statistical Yearbook of Montenegro 2010. Statistical Yearbook 2010 – Podgorica. Available on-line at: <http://www.monstat.org/userfiles/file/publikacije/Statisticki%20godi%C5%A1njak%20CG.-2010,%20za%20WEB.pdf>

(33) Opsteegh M, Swart A, Fonville M, Dekkers L, van der Giessen J (2011) Age-Related Toxoplasma gondii Seroprevalence in Dutch Wild Boar Inconsistent with Lifelong Persistence of Antibodies. PLoS ONE 6(1): e16240. doi:10.1371/journal.pone.0016240

(34) Central Statistical Office of Poland (2009) http://www.stat.gov.pl

(35) Lopes FJV, Borges JMF (2004). Wild Boar in Portugal. Galemys, 16 (spec issue): 243-252

(36) National Institute of Statistics (2009) http://www.insse.ro

(37) СОСТОЯНИЕ ОХОТНИЧЬИХ РЕСУРСОВ В РОССИЙСКОЙ ФЕДЕРАЦИИ В 2008-2010 гг. Информационно-аналитические материалы. // Охотничьи животные России (биология, охрана, ресурсоведение, рациональное использование) Выпуск 9. М.: Физическая культура, 2011. 219 с. Available on-line at: www.mnr.gov.ru/upload/iblock/bf5/ohota_resurses.doc

(38) Statistical Office of the Republic of Serbia (2009) <http://webrzs.stat.gov.rs>

(39) Statistical Office of the Slovak Republic (2009) <http://portal.statistics.sk>

(40) Statistical Office of the Republic of Slovenia (2008) <http://www.stat.si>

(41) Ministerio de Agricultura, Alimentación y Medio Ambiente http://www.marm.es/es/biodiversidad/temas/

(42) Magnusson M (2010) Population and management models for the Swedish wild boar (Sus scrofa) Independent project/ Degree project in Biology • 30 hp • Advanced level D, Natural Resources Programme - Biology and Soil Science • Master´s thesis 2010:18 Swedish University of Agricultural Sciences, The Faculty of Natural Resources and Agricultural Sciences, Department of Ecology, Grimsö Wildlife Research Station, Grimsö

(43) BAFU / OFEV / UFAM (2011) Eidgenössische Jagdstatistik. Bundesamt für Umwelt BAFU. <http://www.wild.uzh.ch>

(45) Yavuz E (2007) Turkey maps its flora and fauna. Today’s Zaman. Feza Gazetecilik A.Ş. 2007 <http://www.todayszaman.com/newsDetail_getNewsById.action?load=detay&link=130371>

(47) SFCoU (2010) Numbers, movement and harvest of wild boar (*Sus scrofa*) in Ukraine. State Fauna Cadastre of Ukraine. I. I. Schmalhausen Institute of Zoology of the National Academy of Sciences of Ukraine, Department of Monitoring and Conservation of Animals. <http://biomon.org/cadastre/2tp-hunting/>

1. FAO (2010). EMPRES Watch – FAO takes a close look at the pig sector in Eastern Europe to better understand the threats of African swine fever. (available at ww.fao.org/docrep/012/ak755e/ ak755e00.pdf.)

   ^ii^ FAO (2012). African Swine Fever (ASF) Recent developments and timely updates - Worrisome dynamics: Steady spread towards unaffected areas could have disastrous impact. In Focus on No. 6. [electronic bulletin]. Rome, FAO (available at http://www.fao.org/docrep/016/ap372e/ap372e.pdf)

   ^iii^ FAO (2013) African swine fever in the Russian Federation: risk factors for Europe and beyond. EMPRES WATCH, Vol. 28, May 2013. Rome (available at http://www.fao.org/docrep/018/aq240e/aq240e.pdf

   ^iv^ ФАО (2014). Африканская чума свиней в Российской Федерации (2007–2012 гг.). ФАО. ЖИВОТНОВОДСТВО И ОХРАНА ЗДОРОВЬЯ ЖИВОТНЫХ. Документ No 178. Рим (available at http://www.fao.org/3/a-i3748r.pdf) [↑](#endnote-ref-1)
