# Supplementary material for: Wild boar mapping using population-density statistics: From polygons to high resolution raster maps
Source: PLoS One. 2018 May 16;13(5):e0193295. doi: 10.1371/journal.pone.0193295 (PMC5955487; doi:10.1371/journal.pone.0193295)
Supplement: S2 Table — (DOCX) [file pone.0193295.s004.docx]

**Supporting Information**

**S2 Table Results of the variogram deconvolution for the three bioclimatic regions and relative charts**

| Region | Model type | Sill | Nugget | Range (km) | MSS Error |
| --- | --- | --- | --- | --- | --- |
| Eastern | Spherical | 0.0091 | 0.0033 | 560.166 | 0.057 |
| Western | Exponential | 0.0307 | 0.043 | 9212.494 | 0.11 |
| Southern | Exponential | 0.0524 | 0 | 328.184 | 0.2 |

| Eastern region | 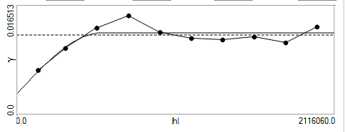 |
| --- | --- |
| Western region | 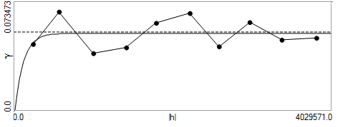 |
| Southern region | 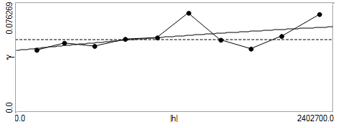 |
