# Supplementary material for: Wild boar mapping using population-density statistics: From polygons to high resolution raster maps
Source: PLoS One. 2018 May 16;13(5):e0193295. doi: 10.1371/journal.pone.0193295 (PMC5955487; doi:10.1371/journal.pone.0193295)
Supplement: S1 Geodataset — (ZIP) [file pone.0193295.s005.zip › Read me file.docx]

**Read me file - Metadata:**

Title: “Predicted wild boar density based on the mosaicked model”.

Columns, Rows: 956, 616

N of Bands: 1

Cellsize (x,y): 5000, 5000

Format: TIFF

Source type: continuous

Pixel Type: floating point

Pixel depth:32 Bit

Extent:

Top 1911481.66514

Left -4582962.03947

Right 197037.96053

Bottom -1168518.33486

Projection: Polar Lambert Azimuthal Equal Area

Linear unit: meter

Datum D_WGS_1984

Source: Pittiglio, Khomenko and Beltran-Alcrudo 2018
